# Comparative evaluation of clinical glycemic control markers treated with imeglimin and its effect on erythrocytes in patients with type 2 diabetes mellitus: study protocol of a single-arm, open-label, prospective, exploratory trial

**DOI:** 10.3389/fphar.2023.1205021

**Published:** 2023-06-07

**Authors:** Takeshi Osonoi, Shinichiro Shirabe, Miyoko Saito, Mitsuru Hosoya, Satako Douguchi, Kensuke Ofuchi, Makoto Katoh

**Affiliations:** Naka Kinen Clinic, Ibaraki, Japan

**Keywords:** imeglimin, HbA1c, glycoalbumin, hemoglobin, erythrocyte lifespan, type 2 diabetes

## Abstract

**Background:** Imeglimin is a novel type 2 diabetes (T2D) drug that is expected to improve mitochondrial function. In its phase 3 clinical trials in Japanese patients with T2D, the hemoglobin A1c (HbA1c) decrease following imeglimin administration was slow, reaching a plateau after 20–24 weeks of treatment. In general, the erythrocyte lifespan may be a factor when HbA1c shows an abnormal value. Therefore, this study will comparatively evaluate HbA1c and other markers of glycemic control in patients with T2D after imeglimin administration and also examine the effects of imeglimin on erythrocytes.

**Methods:** This single-arm, open-label, prospective, exploratory study is designed to evaluate the divergence between HbA1c and glycoalbumin (GA) or 1,5-anhydroglucitol (1,5-AG) and the glycemic reduction rate in 30 patients with T2D with inadequate glycemic control when imeglimin 2,000 mg is administered for 6 months. In addition, we will examine the effect on erythrocytes, the presumed cause of this divergence. We will measure sustained glycemic variability using flash glucose monitoring and examine the relationship between changes in these indices and HbA1c. Moreover, because prolonged erythrocyte lifespan is a possible cause of falsely high HbA1c levels, erythrocyte lifespan, erythrocyte deformability, and hemoglobin concentration will be evaluated as effects of imeglimin on erythrocytes. Furthermore, if imeglimin has an ameliorative effect on erythrocyte deformability, it may improve peripheral arterial disease; thus, we will also evaluate the toe-brachial pressure index, a measure of this effect.

**Discussion:** In this study, if imeglimin administration results in diverging rates of hypoglycemic effect between HbA1c and GA or 1,5-AG and prolongs erythrocyte lifespan, GA and 1,5-AG, rather than HbA1c, will be considered appropriate measures of the hypoglycemic effect in the early stages of imeglimin administration. If imeglimin improves erythrocyte deformability, it may also be a new treatment strategy for peripheral arterial disease, a chronic complication of T2D.

**Ethics and dissemination:** The study protocol was scientifically and ethically reviewed and approved by the Certified Clinical Research Review Board of Toho University (approval number: THU22002). The study protocol was registered in the Japan Registry of Clinical Trials (jRCT) in December 2022 (jRCTs031220489).

## 1 Introduction

The number of patients with diabetes mellitus in Japan has increased with changes in lifestyle and the social environment. A recent survey estimated that there are 10 million people each with diabetes or prediabetes (Ministry of Health, Labour and Welfare, 2017). In addition, diabetes mellitus affects 7.6% of adults aged 20–79 years (Neville et al., 2009), and improving diabetes care is an important issue.

Imeglimin is a drug for type 2 diabetes (T2D) treatment with a novel mechanism launched in Japan in September 2021. Although it is structurally related to metformin, unlike the biguanide class of metformin, imeglimin is a new class drug of tetrahydrotriazine-containing molecules called “glimins” (Theurey et al., 2022). Imeglimin exerts its hypoglycemic effect by promoting glucose-stimulated insulin secretion and improving insulin resistance. Its mechanism of action may be mediated by an effect on the mitochondria (Hallakou-Bozec et al., 2021a), and it may improve mitochondrial function. It has also been suggested that imeglimin acts on the pancreas, skeletal muscle, and liver, three key organs involved in the pathophysiology of T2D, through targeting mitochondria and reducing oxidative stress (Hallakou-Bozec et al., 2021b).

In phase 3 clinical trials (TIMES 1–3) in Japanese patients with T2D, imeglimin, both as a single agent and in combination with other antidiabetic drugs, caused a slow decline in HbA1c that plateaued around 24 weeks after administration (Dubourg et al., 2021; Dubourg et al., 2022; Reilhac et al., 2022). In a retrospective observational study, we reported a slower decrease in HbA1c compared to glycoalbumin (GA) and a divergence in the change rate between the two when imeglimin was administered to patients with T2D (Osonoi et al., 2022a; Osonoi et al., 2022b). HbA1c levels reflect average blood glucose levels over the past 6–12 weeks, which is an indicator of long-term glycemic control, given that the erythrocyte lifespan is approximately 120 days. However, GA, a glycoprotein, is an indicator of short-term glycemic control because the half-life of albumin is shorter than that of erythrocytes at approximately 17–23 days (Takahashi et al., 2007). Therefore, GA accurately indicates changes in blood glucose levels within 2–3 weeks. However, when improvements in blood glucose levels occur in a short period of time, such as with antidiabetic drug treatment, changes in HbA1c are delayed, resulting in a transient divergence between the two. Comparing the rate of decrease from baseline in HbA1c and GA when dipeptidyl peptidase-4 (DPP-4) inhibitors are prescribed, a divergence is seen after 2 months, but thereafter, the two show almost identical trends (Shimizu et al., 2022). However, the divergence between HbA1c and GA observed with imeglimin treatment differed from the results of that report in both magnitude and duration, and short-term changes in blood glucose levels alone cannot explain this (Osonoi et al., 2022a; Osonoi et al., 2022b; Shimizu et al., 2022).

In general, factors that cause abnormal HbA1c values in the divergence between HbA1c and GA include shortening/lengthening of the erythrocyte lifespan (Panzer et al., 1982) and increased/decreased erythrocyte production (Waters et al., 1988). One of the reasons for the higher HbA1c with imeglimin treatment compared to GA may be the prolonged erythrocyte lifespan. However, erythrocyte lifespan testing is not performed in routine clinical practice.

Prolonging the erythrocyte lifespan with imeglimin administration would result in a relatively high HbA1c and a slower rate of glycemic reduction than with GA or 1,5-anhydroglucitol (1,5-AG), suggesting that evaluating the glucose-lowering effect of imeglimin using HbA1c underestimates its effectiveness. There have been no reports, other than ours, on the divergence between HbA1c and GA or 1,5-AG on the glucose-lowering effect of imeglimin in patients with T2D under clinical conditions, or on its effect on erythrocytes (Osonoi et al., 2022a; Osonoi et al., 2022b).

The purpose of the study described in this protocol is to confirm the divergence between HbA1c and the rate of glycemic reduction between GA and 1,5-AG in patients with T2D after treatment with 1,000 mg of imeglimin twice daily for 6 months. In addition, we aim to prospectively examine the effects on erythrocytes, the presumed cause of this divergence.

## 2 Methods/design

### 2.1 Design

The INFINITY study (Comparative evaluation of clinical glycemic control markers treated with ImeglimiN and its eFfect on erythrocytes IN patIents with TYpe 2 diabetes mellitus) is a single-center, single-arm, open-label, prospective study. In clinical practice, the hypoglycemic effect of imeglimin in patients with T2D may differ when measured by HbA1c and GA levels. This study will be conducted in a single-arm, exploratory study (Table 1) because the prolonging of erythrocyte lifespan is postulated as the cause of this divergence. This study will compare HbA1c with the rate of glycemic reduction over time for GA or 1.5-AG to assess whether HbA1c is an appropriate measure. Continuous blood glucose variability measured using flash glucose monitoring (FGM) will also be examined related to changes in these indices to ensure reliability. In addition, because the effect of imeglimin on erythrocyte lifespan is a possible cause of HbA1c deviation, hemoglobin concentration, erythrocyte lifespan, and erythrocyte deformability will also be examined as effects on erythrocytes. Furthermore, if imeglimin ameliorates erythrocyte deformability, it is expected to have the potential to improve peripheral arterial occlusive disease, so an index of arteriosclerosis will also be measured.

**TABLE 1:** Overview of visits and tests schedule.

Item	At informed consent	Pre-observation (months)		Observation period (months)							Follow-up period (months)			At discontinuation		
		−2	−1	0	1	2	3	4	5	6	7	8	9	
Allowable ranges of visits (±weeks)		±2	±2	±2	±2	±2	±2	±2	±2	±2	±2	±2	±2			
Informed consent	X															
Demography	X															
Study drug adherence					X	X	X	X	X	X				X		
Height, body weight				X	X	X	X	X	X	X	X	X	X	X		
Blood pressure, pulse rate				X	X	X	X	X	X	X	X	X	X	X		
Blood tests		X	X	X	X	X	X	X	X	X	X	X	X	X		
Urinalysis				X	X	X	X	X	X	X	X	X	X	X		
Erythrocyte lifespan		X	X	X	X	X	X	X	X	X	X	X	X	X		
Erythrocyte deformability			X	X	X	X	X	X	X	X	X	X	X	X		
Arteriosclerosis/inflammation test				X						X			X	X		
FGM test		X	X	X	X	X	X									
Adverse events		X	X	X	X	X	X	X	X	X	X	X	X	X		

Abbreviations: FGM, flash glucose monitoring.

The observation period will be 6 months as the shortest period to confirm the divergence in the rate of glycemic reduction. The decrease in HbA1c after imeglimin administration is reportedly gradual, reaching a maximum approximately 6 months after the start of administration (Dubourg et al., 2021; Dubourg et al., 2022; Reilhac et al., 2022). In addition, a 2-month pre-treatment observation period will be set to evaluate various conditions before imeglimin administration, and a 3-month follow-up period after administration will be evaluated whether the effects of imeglimin on erythrocytes are reversible. A follow-up period of 3 months will be set to evaluate safety.

The study drug imeglimin (1,000 mg as TWYMEEG ^®^ tablets) will be administered orally twice daily, starting within 1 week after the end of the baseline examination and continuing through the observation period of 6 months. After 6 months, imeglimin will be discontinued, and the patients will be followed for 3 months. The use and dose escalation of an α-glucosidase inhibitor (α-GI) and metformin are allowed for glycemic control during the follow-up period.

The addition of all types of diabetes medications and the escalation of doses of α-GI and metformin taken during the 6-month observation period from the start of imeglimin administration (except in the case of discontinuation) are prohibited because of the possibility that these will affect the evaluation of this study. The use of antiplatelet and antithrombotic drugs is prohibited during the observation and follow-up periods.

The α-GI (excluding acarbose) and metformin used before the day of the consent will be continued in principle without dose change from the day of consent until the end of the 6-month observation period. Drugs deemed medically necessary can be added except for diabetes medications, antiplatelet agents, and antithrombotic agents.

### 2.2 Sample selection

To meet this study’s purpose and ensure the study subject’s safety, patients who meet the following inclusion criteria and do not violate the exclusion criteria will be eligible.

#### 2.2.1 Inclusion criteria

Outpatients who meet all of the following criteria will be eligible:

1. Patients with T2D treated with diet and exercise alone, diet and exercise plus α-GI (excluding acarbose) or metformin, or both drugs in combination
2. Male or menopausal female patients aged ≥20 years on the day of consent
3. Patients with an HbA1c of >6.5% and ≤8.5% on the date of consent
4. Patients with a stable dosage of their diabetes medications from 4 weeks before the day of consent until the day of consent
5. Patients who have provided written informed consent to participate in this study

#### 2.2.2 Exclusion criteria

Patients who fall into any of the following exclusion criteria on the day of consent will be excluded:

1. Patients taking diabetes medications other than α-GI and metformin
2. Patients with anemia (male, <13 g/dL; female <12 g/dL)
3. Patients with hypoalbuminemia (<3.0 mg/dL)
4. Patients with thyroid impairment
5. Patients with liver cirrhosis
6. Patients with nephrotic syndrome or moderate or severe renal impairment (eGFR of <45 mL/min/1.73 m^2^)
7. Patients receiving antiplatelet or antithrombotic drugs
8. Patients diagnosed with malignant tumors
9. Patients requiring a legal representative
10. Patients with a history of hypersensitivity to any ingredients of imeglimin
11. Patients with contraindications to imeglimin
12. Patients with other conditions who are judged by the investigator to be inappropriate for this study

After selecting the potential study participants, the principal investigator or sub-investigators will explain the details of the study using a consent and explanation documents approved by an accredited the Certified Clinical Research Review Board (CRB) of Toho University and obtain their written consent. The principal investigator or sub-investigators will also explain that consent is given freely by the study participant and that the subject will not be unfavorably treated even if he/she does not consent, the participant may withdraw consent at any time after consent is given if he/she changes his/her mind, and that the subject will not be unfavorably treated in such cases. In addition, patients will be withdrawn from the trial if any of the following criteria apply: 1) withdrawal of consent, 2) principal investigator’s decision, based on the patient’s condition, 3) discontinuation of the study or 4) principal investigator’s decision, based on another reason. At the time of discontinuation, confirm the availability of data until discontinuation to the study subjects.

### 2.3 Outcome assessment

#### 2.3.1 Primary outcome

Change in hemoglobin concentration between baseline (month 0) and 6 months after treatment with imeglimin.

#### 2.3.2 Secondary outcomes

Items 1–4 below will be compared between baseline (month 0) and each time point in the pre-observation, observation, and follow-up periods, and between 6 months after imeglimin administration and each time point in the follow-up period. The change from baseline (maximum and mean values) in the pre-observation and observation periods will also be evaluated.

1. Erythrocyte lifespan

Erythrocyte lifespan is calculated by measuring the carbon monoxide (CO) concentration in exhaled breath using a Carbolizer (Taiyo Co., Ltd., Osaka, Japan) and using the Strocchi et al., formula shown below (Strocchi et al., 1992):

Erythrocyte lifespan (days) = K × hemoglobin (g/mL)/endogenous CO (ppm).

K = 1380 (conversion factor).

2. Erythrocyte deformability

Erythrocyte deformability is measured using a blood fluidity measuring device MC-FAN (Optima Inc., Tokyo, Japan). The microchannel array passage time of 100 μL heparinized whole blood is an index for blood fluidity.

3. Red blood cell count, white blood cell count, hemoglobin concentration, and hematocrit (Ht)
4. Corpuscular constant (mean corpuscular volume: MCV, mean corpuscular hemoglobin concentration: MCHC, mean corpuscular hemoglobin content: MCH)

Items 5 and 6 below will be compared between baseline and the pre-observation or observation periods.

5. Measured values of HbA1c, GA, and 1,5-AG, change from baseline, and percent reduction in blood glucose with baseline as 100% (percent reduction in blood glucose will also be compared between each index at each time point)
6. GA/HbA1c and 1,5-AG/HbA1c ratios

For items 7 and 8 below, data measured by the FGM system using FreeStyle Libre Pro (Abbott Diabetes Care Inc., Alameda, CA, United States of America) will be used to compare baseline with each time point during the observation period (up to 3 months after imeglimin administration). FreeStyle Libre Pro should be used following the package insert.

7. Change in mean daily glucose levels and percentage decrease in blood glucose (baseline is the day before imeglimin administration)
8. Mean 14-day glucose levels, estimated HbA1c levels, glucose management indicator (GMI), and respective percentage decreases in glycemia (baseline is 14 days before imeglimin administration):

Estimated HbA1c (%) = (mean glucose [mmol/L] + 2.59)/1.59.

GMI (%) = 3.31 + 0.02392 × (mean glucose [mg/dL])

9. Comparison of measured HbA1c levels and estimated HbA1c values at each time point in the pre- and post-observation periods and the respective percentage reduction in blood glucose at each time point

#### 2.3.3 Safety outcomes

The following items will be compared between baseline (month 0) and each of the observation and follow-up time points for imeglimin:

1. Incidence of adverse events and diseases
2. Liver function markers: measured values of aspartate aminotransferase (AST), alanine aminotransferase (ALT), and gamma glutamyl transpeptidase (γGTP)
3. Lipid markers: measured values of total cholesterol, high-density lipoprotein (HDL)-cholesterol, and triglycerides (TG)
4. Blood pressure, pulse rate, and body weight (measured values of systolic/diastolic blood pressure, pulse rate, and body weight) and calculated value of body mass index (BMI)

#### 2.3.4 Exploratory outcomes

1. Comparison of arteriosclerosis and inflammatory stress markers at baseline (month 0) and 6 months after administration and 3 months after withdrawal of imeglimin:

Arteriosclerosis markers: changes in brachio-ankle pulse wave velocity (baPWV) and toe-brachial pressure ratio (TBI) using a volume-plethysmographic apparatus (BP-203RPEIII; Omron Healthcare, Kyoto, Japan). The baPWV and TBI are measured by trained clinical technicians in a quiet and temperature-controlled clinical measurement room after the patient rested for >5 min in the supine position (Yamashina et al., 2002).

Inflammatory oxidative stress markers: changes in serum high-sensitivity C-reactive protein (hs-CRP) and urinary 8-hydroxydeoxyguanosine (8-OHdG).

2. Stratified analysis and comparison by each antidiabetic drug

### 2.4 Handling of adverse events (AEs)

The INFINITY study will monitor safety by collecting AEs during the study period. An AE is defined as any medically unfavorable events or unintentional injuries or signs (including abnormal changes in laboratory values) that occurs in a study participant. An AE does not necessarily have to be causally related to the study. When an AE occurs, the principal investigator or co-investigators must take immediate and appropriate action and follow up on all patients experiencing the AE until symptoms resolve or clinically significant laboratory abnormalities return to baseline. All AEs will be evaluated and documented on the case report form (CRF). The information that will be collected about AEs will include their date of onset, severity, associated treatment, predictability, outcome, and causation. Additionally, a serious AE is defined as any untoward medical event that occurs at any dose, results in death, a life-threatening condition, requires hospitalization, and results in significant disability or incapacity. All serious AEs will be reported to the principal investigator and sent an e-mail to the manufacturers and distributors of imeglimin (Sumitomo Pharma Co., Ltd.) from the day they learn about serious adverse events to the next day. The principal investigator must report all AEs suspected of having causal relationships with the study to the CRB of Toho University.

### 2.5 Compensation for health hazards

There is no anticipated harm resulting from participation in this study, and participants will not receive compensation for their involvement. It is treated the same as the health hazard and medical accident which occurred in the usual medical care. The drugs prescribed in this study are appropriately used in accordance with the instructions of the doctor in charge, and if serious adverse events occur, they are subject to the application for relief benefits under the drug side effect relief system as in the case of daily medical care. The treatment for other side effects is carried out by insurance medical care. In addition, our clinic shall subscribe to the liability insurance for clinical research as a measure in the event of legal negligence in the case of health damage caused by this study.

### 2.6 Sample size calculation

The target sample size was set to 30.

No data on the effect of imeglimin on erythrocyte lifespan are available; however, in-house data from a Japanese phase 3 study conducted in 27 patients at our clinic showed that imeglimin significantly reduced hemoglobin levels in pre- and post-administration comparisons. Therefore, the sample size was determined based on these data. The mean hemoglobin levels before and 1 year after imeglimin administration were 15.2 and 14.5 g/dL, respectively, with a change of −0.7 g/dL. The standard deviation of the change was 0.8 g/dL; however, in this study, we estimated 1.0 g/dL to account for the study variability. Therefore, the required sample size is 24 when the significance level *α* = 0.05 (two-sided) and the power (1-β) is 90%. Considering dropouts, the target sample size was set to 30. To ensure that enough participants enroll to achieve the target sample size, we have screened potential participants based on inclusion and exclusion criteria, and patients are referred to the study if applicable.

### 2.7 Data collection and management

The principal investigator and sub-investigators will fill in all of each patient’s data in a CRF. It is necessary that all data recorded in the CRF are consistent with the original material, unless data recorded directly in the CRF are used as the source material. During the study, the investigators will collect data at each visit in accord with the schedule in Table 1. All data recorded in the CRF will be checked by the data manager and will then be fixed/set. All study findings and documents will be made confidential, and patients will always be identified by their patient number, never by name. Confidential patient-identifying documents will be maintained by the investigators to preserve participant anonymity. Thus, data management, including coding, security, storage and cleaning, will be performed by investigators throughout the study. These study data (i.e., consent forms, questionnaires, data extracted from practice records) will be stored in a locked cabinet in the investigator’s office and will be archived at Naka Kinen Clinic for 5 years after study completion.

### 2.8 Statistical analysis

Summary statistics of the background data of the study participants will be calculated. For nominal variables, the numbers in each category and their percentages will be shown; for continuous variables, the numbers, mean, standard deviation, minimum, median, and maximum values will be calculated.

Two analysis groups will be used to assess the efficacy of this study: the full analysis set (FAS) and the per-protocol set (PPS), with FAS being the primary analysis. The FAS will be the population of eligible patients who received at least one dose of the study drug and have hemoglobin concentration measurements at baseline and at least one time point during the observation period. The PPS will be the population from the FAS, excluding those who deviated from the study protocol (e.g., use of prohibited concomitant drugs or study drug adherence of <80%). The safety analysis set (SAS) will be used to determine the safety of the study, and the SAS will be the population of patients who received at least one dose of the study drug and have data on any of the safety outcomes.

Summary statistics of hemoglobin levels before and at 6 months after imeglimin, the primary efficacy endpoint, will be calculated. A one-sample *t*-test or Wilcoxon’s signed rank test will be used to evaluate its statistical significance. For the secondary outcomes obtained from the metric data, summary statistics will be calculated for each observation point, a one-sample *t*-test or Wilcoxon’s signed rank test will be performed to evaluate statistical significance, and, if necessary, analysis of covariance, and multivariate analysis will be performed. If the data are non-normally distributed, appropriate data transformations will be applied. When statistical tests are conducted, the significance level will be set at 0.05 (two-sided), with the confidence coefficient for statistical estimation being 95% (two-sided).

In addition, the principal investigator and a biostatistician will confirm the detailed statistical analysis plan before the database is locked. All laboratory data are analyzed by an independent a biostatistician at the implemented in compliance with an outsourcing agreement.

### 2.9 Premature termination of the study

Given the short duration of the study and the minimal risks associated with participation, no interim analysis will be performed in this study. The decision to discontinue the study will be made by the principal investigator based on the following reasons: when a serious violation or non-compliance of the Clinical Research Act or research plan is found; in the case of obtaining a fact that undermines or may undermine ethical or scientific rationality; in the case of a request for discontinuation or recommendation by the administrator of the implementing medical institution, the Ministry of Health, Labour and Welfare, and the CRB; In the case where the principal investigator responsible for the research determines the discontinuation.

### 2.10 Confidentiality

Data containing personal information of the study subjects to be collected in this study will be deleted when transcribing from the electronic medical record to the CRF, and a study subject identification code will be assigned for the study. The study subject identification code is a number that has no regularity with the medical record ID, and the last two digits are assigned in the order of enrollment in the study. In addition, the principal investigator will prepare a list of study subject identification codes to identify individual study subjects and keep it locked in a lockable locker.

### 2.11 Access to data

The investigators must not disclose the information contained in the CRFs to third parties without written approval from the principal investigator, except for regulatory agencies. Only investigators can access data. The datasets used or analyzed will be available from the principal investigator on reasonable request after the study has been completed.

### 2.12 Audit of the study

In order to ensure the reliability of the study results, an audit will be conducted on whether the study was conducted in accordance with Japan’s Clinical Trials Act and research plans. The audit of this study will be independently implemented in compliance with an outsourcing agreement. An independent audit team will inspect Naka Kinen Clinic, the research institute to ensure study data quality.

## 3 Discussion

In this study, the effect of imeglimin administration on lowering blood glucose in type 2 diabetic patients with inadequate glycemic control will be measured using HbA1c, GA and 1,5-AG as indices. The divergence between HbA1c and the rate of blood glucose-lowering with GA and 1,5-AG will be evaluated whether HbA1c is an appropriate index for imeglimin administration. The effect of imeglimin on erythrocytes will also be examined as a cause of the divergence. If imeglimin lengthens erythrocyte lifespan, HbA1c is not an appropriate measure of the hypoglycemic effect of imeglimin in the early stages of administration, but GA and 1,5-AG are appropriate measures to evaluate instead. If imeglimin improves erythrocyte deformability, imeglimin may be a new treatment strategy for peripheral arterial occlusive disease, a chronic complication of T2D.

In the TIMES 1–3 studies, imeglimin, both as monotherapy and in combination with other antidiabetic drugs, slowly decreases HbA1c, reaching a plateau around 24 weeks after administration (Dubourg et al., 2021; Dubourg et al., 2022; Reilhac et al., 2022). Furthermore, the fasting blood glucose trend in the TIMES 1 (monotherapy) study (106 subjects) showed a large decline at week 4 and plateaued from weeks 8 to 24 (Pharmaceuticals and Medical Devices Agency, 2021). This observation differed from the slowly declining trend of HbA1c, which was measured simultaneously. We also reported in a retrospective observational study that, under routine medical care, imeglimin treatment of type 2 diabetic patients resulted in a slower decrease in HbA1c than GA, with a divergence in the rate of change between the two (Osonoi et al., 2022a; Osonoi et al., 2022b). These results suggest that HbA1c may appear to be high. To assess whether HbA1c is an appropriate measure, this study will ensure reliability by comparing HbA1c with the rate of decrease in blood glucose over time for GA or 1.5-AG and by measuring sustained blood glucose variability with FGM and its association with changes in these measures.

In general, HbA1c levels depend not only on blood glucose but also on erythrocyte lifespan. Therefore, shortening or lengthening erythrocyte lifespan results in falsely low or high HbA1c levels, respectively (Panzer et al., 1982; Waters et al., 1988). If HbA1c is falsely elevated by imeglimin, lengthening of erythrocyte lifespan is suggested, but erythrocyte lifespan has not been studied in routine practice. Thus, this study will examine hemoglobin concentration, erythrocyte lifespan, and erythrocyte deformability to determine the effects on erythrocytes. Furthermore, if imeglimin improves erythrocyte deformability, it is expected to have the potential to be effective in treating peripheral arterial occlusive disease, so an index of arteriosclerosis will also be measured.

Erythrocytes can deform and pass through capillaries flexibly (erythrocyte deformability), which improves blood flow through good blood circulation and oxygen transportation (Shigihara et al., 1993). Erythrocyte deformability is mainly dictated by three factors: geometric cell factors (surface area to volume ratio, morphological changes), internal cell viscosity, and cell membrane viscoelasticity (Mohandas et al., 1993). MCV is related to the surface area to volume ratio, and increased MCV improves erythrocyte deformability (von Tempelhoff et al., 2016). In addition, internal erythrocyte viscosity is dependent on hemoglobin concentration; viscosity increases non-linearly with increasing MCHC. and erythrocyte deformability decreases markedly (von Tempelhoff et al., 2016). In our retrospective observational study, imeglimin increased MCV and decreased MCHC (Osonoi et al., 2022b). These results suggest that imeglimin may improve erythrocyte deformability. Various blood rheology abnormalities have been reported in diabetes mellitus, and there are reports of decreased erythrocyte deformability (Shin et al., 2007). Therefore, the ability of imeglimin to improve erythrocyte deformability would greatly benefit patients with T2D.

**FIGURE 1:**
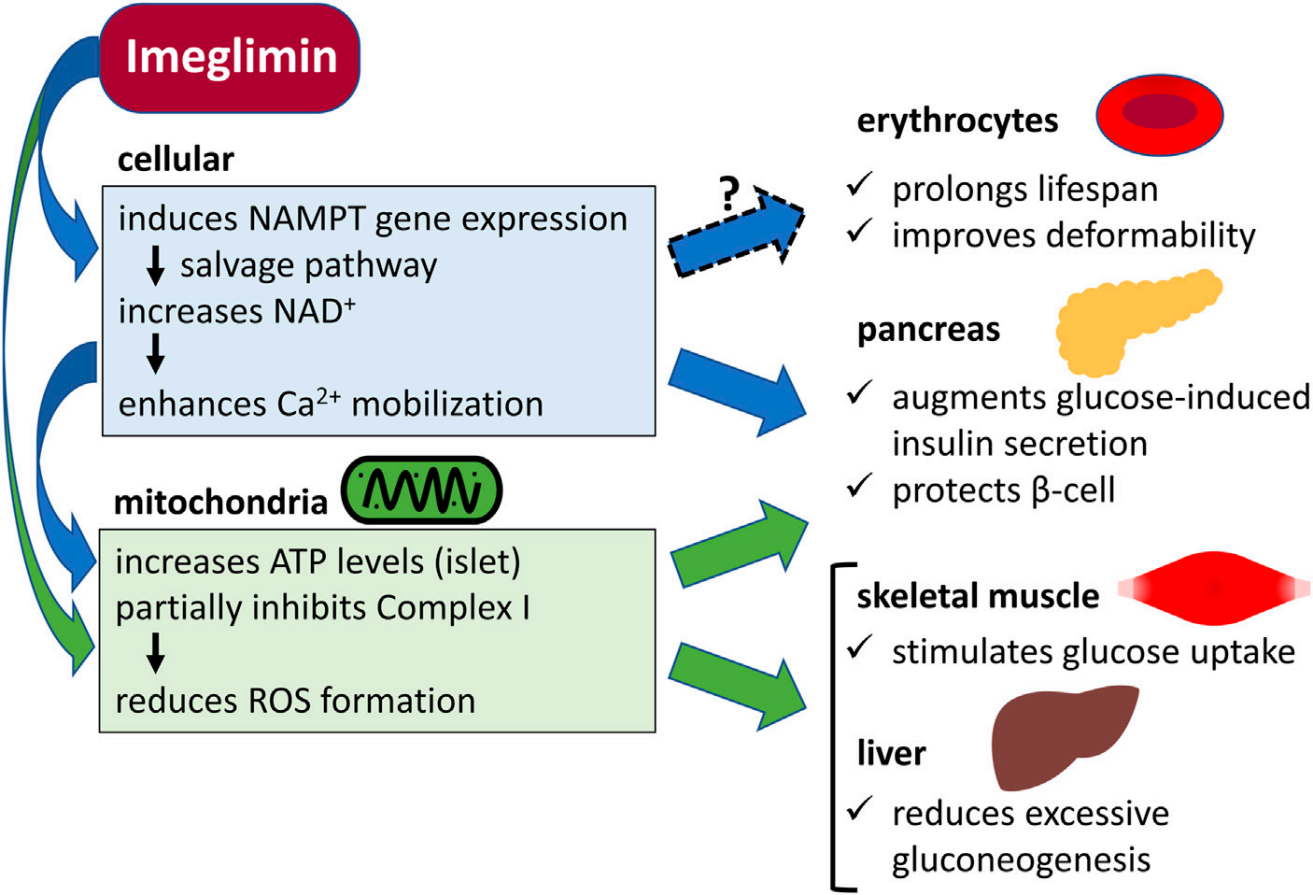
Potential cellular and mitochondrial mechanisms of the action of imeglimin in erythrocytes, pancreas, skeletal muscle, and liver. NAMPT, nicotinamide phosphoribosyltransferase; NAD^+^, nicotinamide adenine dinucleotide; Ca^2+^, calcium ion; ATP, adenosine triphosphate; Complex I, mitochondrial respiratory chain complex I; ROS, reactive oxygen species.

Imeglimin is a drug with a novel mechanism of mitochondrial function improvement. It has been suggested that imeglimin converts nicotinamide (NAM) to NAM mononucleotide (NMN) via increased gene expression of NAM phosphoribosyltransferase (NAMPT) in the salvage pathway utilizing NAM in islet beta cells. Then, NAM adenine dinucleotide (NAD^+^) is synthesized from NMN by NMN adenylyltransferase (NMNAT), which may enhance insulin secretion as well as mitochondrial function (Hallakou-Bozec et al., 2021a). However, NMNAT is also expressed in the cytoplasm of human erythrocytes (Stefano et al., 2010). Mice lacking the NMNAT gene show a marked decrease in erythrocyte NAD^+^ levels and a shorter erythrocyte lifespan of about 10 days compared to that of normal mice (about 60 days), with most of them having deformed and abnormal morphology (Hikosaka et al., 2014; Yaku et al., 2019). Therefore, if imeglimin can increase erythrocyte NAD^+^ levels, it would affect erythrocyte lifespan and deformability (Figure 1).

Although this study is limited by being a small-scale, single-arm, open-label study, it is the first prospective interventional study to evaluate not only the divergence between HbA1c and GA or 1,5-AG on the hypoglycemic effect of imeglimin but also its effect on erythrocytes.

### 3.1 Trial status

The study protocol was fixed on 11 November 2022, and enrollment started on 14 February 2023. Enrollment is expected to be completed by 31 December 2023.

## 4 Ethics and dissemination

Before the study, the study protocol was scientifically and ethically reviewed and approved by the CRB of Toho University (approval number: THU22002). The study protocol was registered in the Japan Registry of Clinical Trials (jRCT) in December 2022 (jRCTs031220489). This study will be conducted at the Naka Kinen Clinic in accordance with the ethical principles of the Declaration of Helsinki and in compliance with the “Clinical Research Act.” The physician in charge of the study will provide sufficient explanation to the study participants using the consent and explanation documents and will obtain their free and voluntary consent in writing. The results of this study will be published through submission to a peer-reviewed scientific journal.

## Data Availability

The original contributions presented in the study are included in the article/supplementary material further inquiries can be directed to the corresponding author.
